# Evaluating the Resilience of ICU Nurse Staffing Standard Operating Procedures Under Demand Variability: A Discrete Event Simulation Study Using MIMIC-IV

**DOI:** 10.3390/healthcare14152344

**Published:** 2026-08-01

**Authors:** Jomana Bashatah, Adel Bashatah

**Affiliations:** 1 Department of Industrial and Systems Engineering, College of Engineering, Princess Nourah Bint Abdulrahman University, Riyadh 11671, Saudi Arabia; 2 Department of Nursing Administration and Education, College of Nursing, King Saud University, Riyadh 11451, Saudi Arabia; abashatah@ksu.edu.sa

**Keywords:** intensive care unit, nurse staffing, standard operating procedures, discrete event simulation, patient safety, healthcare systems engineering, MIMIC-IV, workforce resilience, adverse events, workload modeling

## Abstract

**Background/Objectives**: ICU nurse staffing Standard Operating Procedures (SOPs) govern nurse-to-patient assignment and escalation rules, yet their robustness under demand variability and workforce disruption has not been quantitatively evaluated. **Methods**: A Discrete Event Simulation model of a 20-bed ICU was parameterized from 20,419 MICU stays extracted from MIMIC-IV v3.1. Three SOPs—Fixed Ratio, Acuity-Adjusted, and Dynamic Escalation—were evaluated across four scenarios (baseline, census surge, nurse shortage, combined disruption), each with 100 replications, using Kruskal–Wallis and Dunn’s post hoc tests. **Results**: Dynamic Escalation achieved the lowest adverse event rate at baseline (4.25 per 100 patient days), a 16.8% reduction relative to Fixed Ratio (5.11). However, a resource-constrained comparison equalizing total nurse hours across protocols reversed this ranking: Dynamic Escalation performed significantly worse than both static protocols (*p* < 0.0001), which were themselves statistically indistinguishable, indicating that its primary advantage stems from greater staffing capacity rather than superior decision logic. **Conclusions**: Apparent differences between ICU staffing SOPs are largely driven by total available nurse hours rather than allocation logic. Investment in staffing capacity, rather than a specific allocation rule, may be the primary lever for improving ICU patient safety outcomes.

## 1. Introduction

Intensive care units (ICUs) represent the most resource-intensive environment in modern healthcare, where critically ill patients require continuous, high-acuity nursing care. The organizational structure governing how nursing resources are allocated in these settings is largely codified through Standard Operating Procedures (SOPs), which are formally defined protocols that specify nurse-to-patient assignment rules, task prioritization hierarchies, and escalation thresholds. According to the Systems Engineering Initiative for Patient Safety (SEIPS) model, the structural and organizational characteristics of healthcare work systems, including how staffing protocols are designed and enforced, are primary determinants of care quality and patient safety outcomes [1]. While SOPs are intended to standardize care delivery, their adequacy under conditions of real-world demand variability, patient acuity fluctuation, and workforce shortages remains insufficiently evaluated from a quantitative systems engineering perspective [2].

The relationship between nurse staffing levels and patient outcomes is well-established and continues to attract significant policy attention. Recent multicenter evidence from over 183,000 adult inpatient admissions demonstrates that nurse staffing coverage is significantly associated with in-hospital mortality, 30-day hospital readmission, and nurse-sensitive adverse events including healthcare-acquired infections [3]. The strength of this evidence has driven legislative action, including the 2023 New York ICU staffing law, which mandates a minimum of one registered nurse per two ICU patients, adjusted upward based on patient acuity, in recognition that fixed ratios are insufficient under variable clinical conditions [4]. Simultaneously, a multinational study across 10 countries found that the majority of ICUs present persistently high nursing workload, with patient-to-nursing staff ratios varying from 0.8:1 to 1.9:1, and that nursing staff hours exceeded workload requirements in 70% of surveyed countries, underscoring the global inconsistency of current staffing practices [5]. In this study, robustness under disruption is operationalized as the capacity of an ICU staffing protocol to maintain acceptable patient safety performance under demand variability and workforce disruption, measured as the relative change in adverse event rate from baseline to disrupted operating conditions.

A critical limitation of the existing research is its near-exclusive reliance on observational study designs that document associations between staffing levels and outcomes, without providing a causal, prospective evaluation of the protocols that govern staffing decisions. ICU nursing workload is persistently high and unevenly distributed, yet staffing levels are rarely adjusted to reflect actual care demands [6]. The Nursing Activities Score (NAS), one of the most widely validated tools for quantifying ICU nursing workload at the task level [6], reveals that standard fixed-ratio SOPs frequently fail to account for within-shift acuity variability, a structural flaw that cannot be detected through aggregate ratio analysis alone. Furthermore, alternative models of nurse staffing that deviate from evidence-based protocols have been characterized as potentially dangerous in high-stakes hospital care [7], yet the comparative safety profile of different SOP configurations under disruption scenarios has not been systematically quantified.

From a systems engineering standpoint, ICU nursing workload can be understood as a quantifiable, dynamic system property. Systems engineering analyses of ICU operations have shown that patient census and acuity characteristics are measurable workload indicators that directly constrain care team cognitive capacity and clinical decision-making [8], establishing a rigorous quantitative foundation for simulation-based workload modeling. The impact of nurse staffing on patient safety is further compounded during periods of demand disruption: higher nurse-to-patient ratios have been associated not only with adverse patient outcomes but also with nurse burnout, job dissatisfaction, and increased turnover intent [9], creating a compounding systemic risk that fixed-ratio SOPs are structurally unable to address.

Discrete Event Simulation (DES) offers a rigorous and ethically sound methodology for evaluating staffing SOPs under conditions that cannot be tested in real clinical environments. DES is well-suited to modeling the stochastic, queue-driven dynamics of ICU patient flow, allowing researchers to stress-test alternative SOP configurations across a range of demand scenarios without risk to real patients [10]. Recent applications have demonstrated its utility in emergency department nurse staffing optimization [11], patient flow management [11], and ICU capacity planning [8]. Notably, recent work combining AI-based demand forecasting with DES has shown that merging predictive modeling with simulation can identify conditions under which current nurse staffing is insufficient to ensure timely patient treatment [11], a finding that motivates extending this approach to SOP evaluation in the ICU context. Despite this growing body of work, no study has used DES to systematically compare the robustness under disruption of alternative ICU staffing SOP designs across multiple disruption scenarios, representing a gap this study directly addresses.

This study develops a Discrete Event Simulation model of an ICU unit, parameterized using clinical data from MIMIC-IV v3.1 [12], a publicly available electronic health record database covering over 94,000 ICU admissions, to evaluate three SOP configurations: (1) a fixed nurse-to-patient ratio protocol, (2) an acuity-adjusted assignment protocol, and (3) a dynamic escalation protocol. Each SOP is stress-tested across four demand scenarios: normal operations, patient census surge, nurse workforce shortage, and a combined disruption condition. The principal contribution is a quantitative, systems-level comparison of SOP robustness under disruption, defined as the capacity to maintain safe patient outcomes under demand variability, providing directional, simulation-based guidance for ICU staffing policy design.

## 2. Materials and Methods

### 2.1. Study Design

This study employs a Discrete Event Simulation (DES) approach to model ICU operations and evaluate the performance of three nurse staffing Standard Operating Procedures (SOPs) under varying demand conditions. The study follows a four-phase methodology: (1) data extraction and parameterization from the MIMIC-IV clinical database, (2) conceptual model development and SOP formalization, (3) simulation model implementation and validation, and (4) scenario analysis with performance metric computation. Model development and reporting adhere to the STRESS (Strengthening the Reporting of Empirical Simulation Studies) guidelines to ensure transparency and reproducibility [13].

### 2.2. Data Source and Cohort Definition

Patient flow parameters were extracted from MIMIC-IV version 3.1, a large-scale, publicly accessible, de-identified electronic health record database sourced from the Beth Israel Deaconess Medical Center (BIDMC) in Boston, MA, USA [12]. MIMIC-IV v3.1 encompasses ICU stays from 2008 through 2022, covering over 94,000 hospitalizations of critically ill adult patients. Data were accessed via Google BigQuery following completion of the required Collaborative Institutional Training Initiative (CITI) credentialing process through PhysioNet.

The study cohort was defined as all ICU stays in the Medical Intensive Care Unit (MICU) with a length of stay between 4 and 720 h, yielding a final cohort of 20,419 qualifying MICU stays across 14 years of data. The MICU was selected as the target unit type because it represents the most common adult medical ICU configuration in the United States, with a median size of 20 beds, directly matching the modeled unit capacity [14]. Restricting the cohort to a single unit type ensures that the extracted arrival rates, length of stay distributions, and acuity proportions reflect the operational characteristics of the specific unit being modeled rather than an aggregate across heterogeneous unit types. The 4 h lower bound excludes administrative errors and very short observation stays; the 720 h upper bound excludes extreme outliers representing fewer than 0.5% of stays.

Patient acuity was classified using first-day Sequential Organ Failure Assessment (SOFA) scores extracted from the mimiciv_3_1_derived.sofa table, using the maximum SOFA score recorded within the first 24 h of each ICU stay. Patients were stratified into three acuity tiers—low (SOFA 0–5), moderate (SOFA 6–10), and high (SOFA ≥ 11)—yielding a final MICU cohort of 20,419 stays with a daily admission rate of 3.99 patients per day.

### 2.3. Model Parameterization

Simulation input parameters were derived from two sources, as summarized in Table 1: patient flow parameters (arrival rates, length of stay distributions, and acuity proportions) were extracted directly from the MIMIC-IV cohort, while nursing task demand rates, adverse event probabilities, and workload thresholds were calibrated from published NAS-based workload studies, as direct nursing task timestamps are not reliably extractable from MIMIC-IV chart events at the required granularity [6,15]. Specifically, the adverse-event probabilities (0.008 normal workload, 0.035 overloaded) were calibrated so that simulated baseline adverse-event rates fell within the 2–5 events per 100 patient days range reported in NAS-based ICU workload studies [6,15], rather than derived through direct statistical transformation of NAS measurements, as the source literature reports workload–risk relationships as threshold ranges rather than as parametrized response functions. The overload threshold (0.88) and escalation trigger (0.85) were selected directly from the 85–90% NAS workload range identified in [6] and [15] as the point above which missed-care and adverse-event rates measurably increase.

Patient arrival rates were computed from ICU admission timestamps stratified by hour of day. Contrary to initial assumptions, ICU admissions peak during evening and night hours (18:00–07:00), with an average arrival rate of 0.205 patients/h, compared to 0.121 patients/h during the day shift (07:00–18:00). This pattern reflects the predominance of emergency transfers and post-operative admissions in the evening hours and is consistent with published analyses of ICU admission timing [12]. The overall daily admission rate across the cohort was 3.99 admissions/day.

Length of stay distributions were fitted to log-normal distributions stratified by acuity tier using the method of moments, yielding the following parameters: low acuity (median = 37.0 h, mean = 62.12 h; log-normal: μ = 3.611, σ = 1.018); moderate acuity (median = 70.0 h, mean = 111.96 h; log-normal: μ = 4.249, σ = 0.969); high acuity (median = 107.0 h, mean = 153.23 h; log-normal: μ = 4.673, σ = 0.848).

Acuity distribution derived from SOFA scores yielded: low acuity 64.4% (*n*= 13,149), moderate acuity 27.4% (*n* = 5593), and high acuity 8.2% (*n* = 1677).

Nursing task demand rates and adverse event probability thresholds were calibrated from NAS-based workload studies, as direct nursing task timestamps are not reliably extractable from MIMIC-IV chart events at the required granularity [6,15].

No iterative calibration procedure was performed. All parameters were fixed prior to any simulation experiments based solely on the empirical MIMIC-IV extractions and the published NAS-based values described above. The parameterization fidelity assessment in Section 2.9 was conducted after parameter fixation to verify implementation correctness, not to adjust parameters toward target outputs. This approach ensures that the simulation results are not influenced by circular fitting to the source data.

### 2.4. ICU System Conceptual Model

The ICU was modeled as a closed queueing system with a fixed bed capacity of 20 beds, consistent with median adult medical ICU sizes reported in the literature [14]. The system comprises the following entities, resources, and processes:

- Entities: Individual patients, each characterized by an acuity tier assigned probabilistically at arrival according to the MIMIC-IV-derived acuity distribution.
- Resources: Registered nurses (RNs), modeled as finite shared resources whose availability is governed by the active SOP and shift schedule. Beds are modeled as a secondary resource. Patients who arrive when all beds are occupied wait up to two hours for a bed to become available; if no bed is freed within this window, the patient is redirected to an alternative unit, consistent with standard ICU diversion practice [14].
- Process flow: Patients arrive according to a non-homogeneous Poisson process with shift-specific arrival rates derived from MIMIC-IV. Upon arrival, each patient is assigned an acuity tier, queues for a bed, and upon admission is assigned to nursing care under the active SOP. Patients are discharged or transferred following a length of stay drawn from the acuity-stratified log-normal distributions fitted to MIMIC-IV data.
- Adverse Event Generation: Nurse-sensitive adverse events (medication errors, missed assessments, pressure injuries) are modeled as stochastic Bernoulli events evaluated once per 8 h nursing shift per patient, with probability conditioned on whether the current unit-level nursing workload index exceeds the overload threshold. This shift-level check is consistent with NAS-based evidence that adverse events are attributable to cumulative shift workload rather than individual task failures [15,16]. The 8 h interval was selected specifically because the Nursing Activities Score, from which the adverse event probabilities (0.008 and 0.035) were calibrated, is a shift-level measurement instrument that quantifies cumulative nursing workload over a complete shift rather than at individual task level. Evaluating adverse events at this same interval is therefore consistent with the measurement scale of the underlying data.

**Figure 1:**
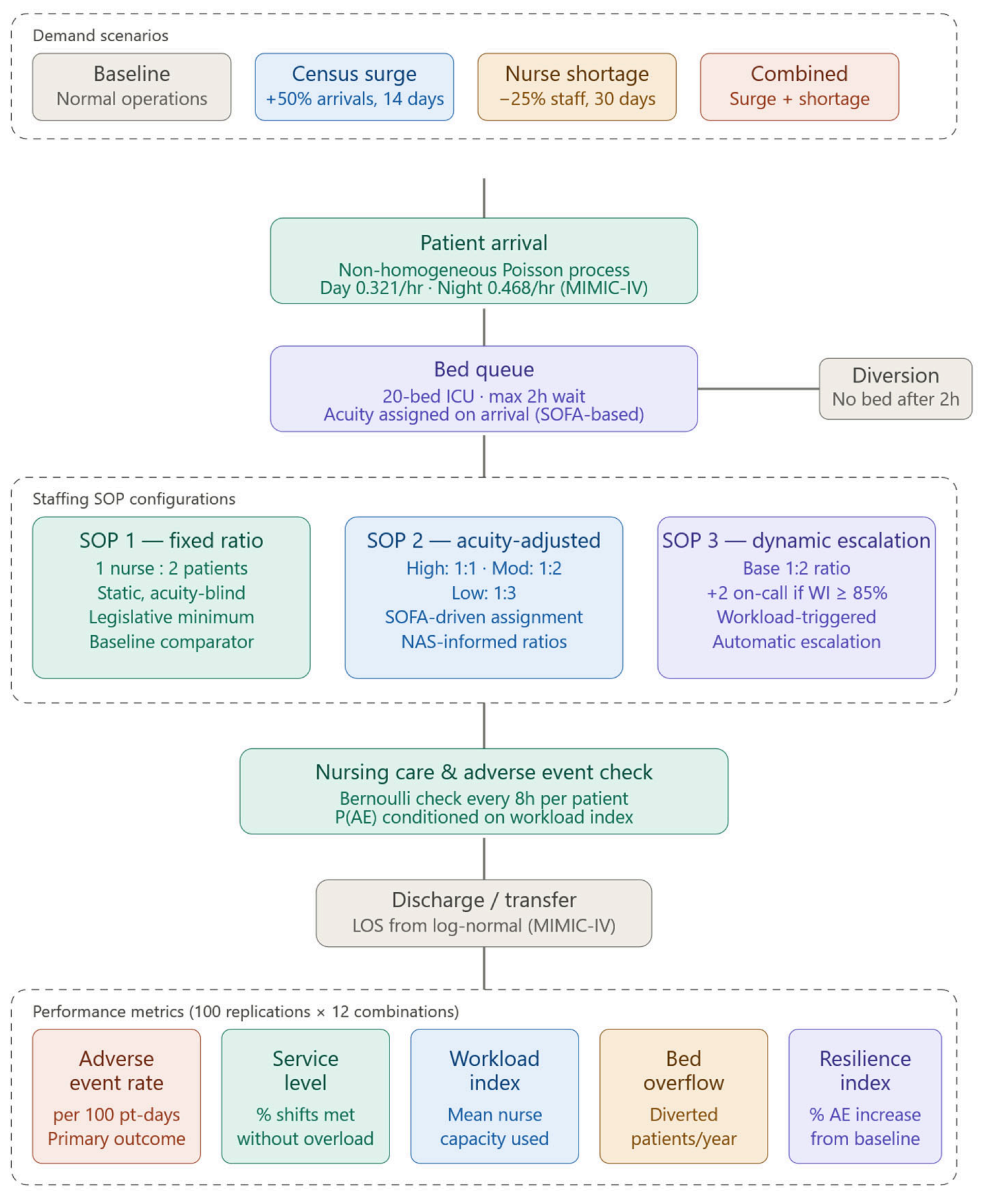
Discrete Event Simulation framework for evaluating ICU nurse staffing SOPs under demand variability. Patient arrivals are generated under four demand scenarios and routed through a 20-bed ICU system governed by one of three SOP configurations. Performance is evaluated across five metrics over 100 replications per scenario–SOP combination.

The complete simulation framework, including patient flow, SOP decision logic, and output metrics, is depicted in Figure 1.

The key modeling assumptions underlying the simulation, together with their rationale, data source, and potential directional impact on results, are summarized in Table 2.

### 2.5. SOP Formalization

Three staffing SOP configurations were formalized and implemented as decision logic within the simulation model:

SOP 1—Fixed Ratio Protocol: A static nurse-to-patient assignment rule maintaining a 1:2 ratio at all times regardless of patient acuity or census fluctuation. This reflects the minimum standard mandated by recent ICU staffing legislation [4] and serves as the baseline comparator.

SOP 2—Acuity-Adjusted Protocol: Nurse-to-patient ratios are dynamically adjusted based on each patient’s acuity tier at the time of bed assignment: 1:1 for high-acuity patients (SOFA ≥ 11), 1:2 for moderate-acuity patients (SOFA 6–10), and 1:3 for low-acuity patients (SOFA 0–5). This SOP operationalizes the NAS-informed principle that staffing levels should reflect actual care demand rather than census alone [5].

SOP 3—Dynamic Escalation Protocol: Maintains the fixed 1:2 baseline ratio but incorporates an automatic escalation trigger: when unit-level aggregate nursing workload exceeds 85% of capacity (measured as total active nursing tasks relative to available nurse hours per shift), two on-call nurses are activated for the remainder of the shift. The overload threshold of 0.88 was selected as the midpoint of the 85–90% workload capacity range reported in NAS-based studies as the level at which nurse-sensitive adverse event rates increase significantly [6,16]. The escalation trigger of 0.85 was set slightly below the overload threshold to allow Dynamic Escalation to activate preventively, i.e., before adverse events begin accumulating, consistent with the safety engineering principle of early-warning intervention. The sensitivity analysis in Section 3.4 varies the overload threshold by ±25% (range: 0.660–0.95) and confirms that the SOP ranking is stable across this full range, with the exception of the boundary condition at 0.660, which is considered implausibly low for real ICU operations. While alternative threshold values have been proposed in the literature, ranging from 80% in some NAS-based studies to 95% in others depending on unit type and acuity mix, the sensitivity analysis in Section 3.4 demonstrates that the SOP ranking is stable across the full range of ±25% variation (0.660–0.95), encompassing the breadth of published threshold values and confirming that conclusions are not dependent on the specific threshold selected.

### 2.6. Scenario Design

Each SOP was evaluated under four demand scenarios designed to represent the operational range of ICU conditions:

Scenario 1—Baseline: Normal operations with arrival rates and LOS distributions derived directly from MIMIC-IV without modification, representing typical steady-state ICU functioning.

Scenario 2—Patient Census Surge: Patient arrival rate increased by 50% for a continuous 14-day period beginning on simulation day 60, simulating a prolonged demand surge consistent with epidemic conditions or regional mass casualty events [11].

Scenario 3—Nurse Workforce Shortage: Available nurse staffing reduced by 25% for a continuous 30-day period beginning on simulation day 60, simulating an extended staffing crisis consistent with pandemic-era workforce attrition and structural nursing shortages documented in the literature [5,17]. Because nurse counts must be represented as whole individuals, the 25% reduction is implemented as max(1, floor(base_nurses × 0.75)), truncating rather than rounding. The two-nurse on-call pool available to Dynamic Escalation is unaffected by the shortage scenario and remains fully available for escalation regardless of base staffing reduction.

Scenario 4—Combined Disruption: Simultaneous application of Scenarios 2 and 3, representing the worst-case co-occurrence of demand surge and workforce shortage, a scenario observed during the COVID-19 pandemic peak periods [17].

### 2.7. Simulation Implementation

The DES model was implemented in Python 3.11 using the SimPy 4.1.1 discrete-event simulation library, selected for its process-interaction paradigm, which is well-suited to modeling concurrent patient and nurse resource interactions. Statistical distributions were fitted using SciPy 1.12.0. Each of the 12 SOP–scenario combinations was replicated 100 times using independent random seeds to obtain stable output distributions, following established DES best practices for replication determination [13]. Replication sufficiency was assessed by monitoring the coefficient of variation (CV) of the primary outcome, adverse event rate per 100 patient days, across sequential replication sets of 25, 50, 75, and 100. At 100 replications, CV fell below 4% across all 12 SOP–scenario combinations, indicating that output distributions had stabilized and additional replications would not materially change the reported means or standard deviations. The narrow 95% confidence intervals reported in Section 3.1 further confirm the stability of estimates at this replication count. A simulation warm-up period of 30 days was applied to eliminate initialization bias, followed by a data-collection horizon of 365 simulated days per replication. The 30-day warm-up was selected as approximately five times the maximum mean length of stay in the model—for example, for high-acuity patients, mean LOS = 152 h ≈ 6.3 days, following the established DES convention of setting the warm-up period to exceed the longest system transient by a factor of at least three to five [13]. This ensures that bed occupancy, nurse workload distributions, and patient acuity mix reach steady-state conditions before data collection begins, eliminating the artificial under-occupancy present at simulation initialization.

### 2.8. Resource-Constrained Comparison

To address the structural non-equivalence of staffing resources across SOP configurations, a supplementary resource-constrained comparison was conducted in which all three SOPs were allocated identical maximum nurse hours. Under this design, Dynamic Escalation’s base staffing was reduced to eight nurses on the day shift and six nurses on the night shift, such that the on-call activation of two nurses restores total capacity to 10 days and 8 nights, equal to the Fixed Ratio and Acuity-Adjusted base staffing. This design isolates the contribution of the escalation decision logic from the resource advantage while maintaining all other model parameters, scenarios, and replications identical to the primary analysis.

### 2.9. Model Parameterization Fidelity

Parameterization fidelity was assessed by comparing the statistical properties of simulation outputs under baseline conditions against the corresponding MIMIC-IV cohort statistics used to derive the input parameters (Table 3). This comparison demonstrates that the implemented log-normal distributions and Poisson arrival processes accurately reproduce the empirical distributions from which they were fitted, confirming implementation correctness. Six of the nine metrics achieved error rates below 1%, with two metrics, daily admissions (6.5%) and high-acuity median LOS (8.9%), showing larger but still acceptable deviation, attributable to simulation stochasticity and the right-skewed tail of the empirical high-acuity LOS distribution. As the simulation parameters are derived directly from MIMIC-IV rather than from an independent dataset, this assessment reflects parameterization fidelity rather than predictive external validity. Independent external validation would require a held-out dataset from a different institution, which is deferred to future work.

As a further check on internal consistency, arrivals, admissions, diversions, and discharges reconcile across all twelve SOP–scenario combinations under the frozen model. At baseline, annual arrivals (1383.8) equal the sum of admissions (1357.3) and diversions (26.5); discharges (1357.7) closely track admissions, consistent with steady-state operation over the 365-day collection window. Under Census Surge and Combined Disruption, arrivals rise to 1411.2/year with diversions increasing to 37.1, while Nurse Shortage alone leaves arrival and diversion counts unchanged from the baseline, as expected since this scenario does not modify patient demand. Annualized patient days (4633.4 at baseline; 4731.8 under surge and combined scenarios) correspond to an average census of 12.7–13.0 patients, consistent with the 68.9–70.4% occupancy reported in Section 3.1, and available nurse hours ranged from 76,330 to 81,868 per year depending on SOP and scenario, with Dynamic Escalation activating on-call nurses on approximately 385–410 occasions annually.

### 2.10. Performance Metrics

System performance under each SOP–scenario combination was evaluated using the following metrics:

- Service Level: Proportion of 8 h shift checks in which nursing care demand was met without workload overload.
- Adverse Event Rate: Number of nurse-sensitive adverse events per 100 patient days; the primary outcome measure.
- Nurse Workload Index: Mean fraction of available nurse capacity consumed by active patient care demand, sampled at each shift check.
- Bed Overflow Count: Number of patients diverted after exceeding the 2 h bed wait threshold.
- Robustness Under Disruption Index: Operationalized as the relative increase in adverse event rate from baseline to each disruption scenario, expressed as a percentage. This operationalization captures the robustness dimension of robustness under disruption, i.e., the ability to resist performance degradation under perturbation, and does not address the adaptation, recovery, learning, or reorganization dimensions recognized in the broader healthcare robustness under disruption engineering literature. It was selected for its direct connection to patient safety outcomes and its computational tractability within the simulation framework.

Statistical comparison of SOP performance across scenarios was conducted using the Kruskal–Wallis test, with Dunn’s post hoc pairwise comparisons and Bonferroni correction for multiple comparisons, applied to the distributions of replicated adverse event rates and service levels across all 100 replications.

## 3. Results

### 3.1. Descriptive Results

Simulation outputs across all 12 SOP–scenario combinations are summarized in Table 4. Across all scenarios, Dynamic Escalation consistently produced the lowest adverse event rates. Under baseline conditions, mean adverse event rates were 4.260 ± 0.478 per 100 patient days for Dynamic Escalation, 5.119 ± 0.568 for Fixed Ratio, and 5.221 ± 0.609 for Acuity-Adjusted, a 16.8% reduction for Dynamic Escalation relative to Fixed Ratio and an 18.4% reduction relative to Acuity-Adjusted. Service levels were highest for Dynamic Escalation (98.66%) followed by Fixed Ratio (98.39%) and Acuity-Adjusted (98.36%). Mean nursing workload indices were lower under Dynamic Escalation (0.720) compared to Acuity-Adjusted (0.751) and Fixed Ratio (0.764), reflecting more efficient capacity utilization through on-call activation. Baseline bed occupancy was 68.9% across all SOPs, consistent with published ICU benchmarks of 65–85%, and annual overflow was 27 patients (approximately 2% of arrivals), a rate consistent with standard ICU diversion practice.

To contextualize the clinical magnitude of these differences, the adverse event rate reduction associated with transitioning from Fixed Ratio to Dynamic Escalation (0.859 per 100 patient days at baseline) corresponds to approximately 40 fewer modeled adverse events per 20-bed ICU per year under baseline conditions, computed as 0.859/100 pd × 4633.4 simulated patient days/year ≈ 40 events, where annual patient days is derived from the flow-accounting reconciliation (Section 2.9). These figures represent model-based estimates conditional on the assumed adverse event probabilities and should be interpreted as illustrative of relative magnitude rather than as precise clinical projections.

Bed overflow counts were identical across SOP configurations within each scenario (27 patients/year at baseline; 37 under surge scenarios), representing approximately 2% of arriving patients, consistent with standard ICU diversion practice. This confirms that overflow is driven by bed capacity constraints rather than nursing protocol design, and that staffing SOPs alone cannot address structural capacity shortfalls.

Workload index remained relatively stable across demand scenarios for all three SOPs (Table 5), ranging from 0.0228 (Dynamic Escalation) to 0.0247 (Acuity-Adjusted) across the four scenarios tested, indicating that none of the three protocols operates near a structural capacity ceiling under the modeled conditions. Dynamic Escalation maintained the lowest workload index throughout (0.7202–0.7430), consistent with its access to additional on-call capacity, while Fixed Ratio and Acuity-Adjusted showed comparable, moderately higher workload levels (0.7511–0.7880 and 0.7643–0.7880 respectively).

### 3.2. SOP Performance During and After Demand Disruption

Because robustness under disruption indices calculated as a percentage change from each protocol’s own baseline can misleadingly favor a protocol operating near its own performance ceiling, and because annualizing a 14- or 30-day disruption over a 365-day simulation dilutes its effect, we report absolute adverse event rate changes during the actual disruption window, using each SOP’s own pre-disruption period as a common reference point (Table 6). We refer to this outcome as robustness under disruption rather than robustness under disruption, as our design measures the magnitude of performance degradation during a disruption but does not model adaptation, learning, or recovery dynamics.

During the 14-day Census Surge window, adverse event rates increased substantially relative to the immediately preceding period—+3.104 per 100 patient days for Fixed Ratio, +2.498 for Acuity-Adjusted, and +1.669 for Dynamic Escalation—a materially larger effect than the annualized figures previously reported suggest, and one that reverses the earlier finding that Fixed Ratio was most resilient: in absolute terms, Dynamic Escalation shows the smallest degradation under every disruption type tested. A parallel pattern held under the 30-day Nurse Shortage window (+2.960/+2.584/+2.017) and the acute phase of Combined Disruption (+5.739/+5.384/+5.227). In all cases, adverse event rates returned to pre-disruption levels immediately in the post-disruption window, with no evidence of lingering elevation or delayed recovery, consistent with treating this outcome as robustness rather than robustness under disruption in the fuller sense used in the healthcare robustness under disruption engineering literature.

To compare disruption types at equal duration, we directly examined the 14-day Census Surge and a symmetric 14-day Nurse Shortage window (Table 7). For Fixed Ratio and Acuity-Adjusted, the two disruption types produced statistically indistinguishable degradation (differences of +0.050 and −0.005 per 100 patient days respectively, well within replication variability), indicating no reliable difference between surge and shortage for these protocols at equal duration. Only Dynamic Escalation showed a consistent, if modest, difference, with shortage producing somewhat greater degradation than surge (+1.777 vs. +1.669). We therefore restrict the conclusion that workforce shortage poses greater risk than census surge to Dynamic Escalation specifically, rather than treating it as a general finding across staffing protocols. The larger effect observed under the full 30-day shortage window (+2.017 for Dynamic Escalation) reflects the accumulated duration of sustained understaffing rather than a difference in disruption type.

### 3.3. Statistical Testing

Kruskal–Wallis tests confirmed statistically significant differences in adverse event rates across SOP configurations under all four demand scenarios (H = 104.106–122.685, *p* < 0.001 for all comparisons), indicating that the SOP design has a robust and consistent effect on patient safety outcomes regardless of operating conditions (Table 8).

Dunn’s post hoc pairwise comparisons with Bonferroni correction confirmed that Dynamic Escalation differed significantly from both Fixed Ratio and Acuity-Adjusted under baseline and combined disruption conditions (*p* < 0.0001 for both pairs, Table 9), while Fixed Ratio and Acuity-Adjusted did not differ significantly from each other at either baseline (*p* = 0.81) or combined disruption (*p* = 1.00). Effect size analysis using the rank-biserial correlation confirmed moderate-to-large practical differences for Dynamic Escalation relative to the static protocols—Fixed Ratio vs. Dynamic Escalation (r = −0.745 baseline, −0.717 combined) and Acuity-Adjusted vs. Dynamic Escalation (r = −0.781 baseline, −0.726 combined)—while Fixed Ratio vs. Acuity-Adjusted showed negligible effect sizes (r = 0.090 baseline, 0.070 combined), consistent with the absence of a significant difference between these two protocols.

These effect sizes should be interpreted as confirming a real structural difference between Dynamic Escalation and the two static protocols within the model, attributable to Dynamic Escalation’s access to additional on-call nurse hours (Section 3.4 and Section 3.5, resource-constrained comparison), rather than as estimates of clinical effect sizes in real ICU populations, where confounding, implementation variability, and patient heterogeneity would substantially attenuate observed differences. The workload index difference underlying this separation is modest in absolute terms (Fixed Ratio WI = 0.764 vs. Dynamic Escalation WI = 0.720 at baseline; Table 5), consistent with a real but bounded effect rather than the near-complete distributional separation suggested by effect sizes closer to ±1.0. Readers should therefore weigh the absolute adverse-event-rate differences and workload evidence reported in Section 3.1, together with the resource-constrained comparison in Section 3.5, more heavily than statistical significance alone in interpreting the practical implications of this study.

### 3.4. Sensitivity Analysis

To assess the robustness of the SOP ranking to the three literature-derived adverse event parameters: baseline adverse event probability (0.008), overloaded adverse event probability (0.035), and overload workload threshold (0.88), a sensitivity analysis was conducted varying each parameter individually by ±25% while holding the others constant (Table 10). Across all five parameter configurations and both the baseline and combined disruption scenarios, the SOP ranking remained completely stable: Dynamic Escalation consistently produced the lowest adverse event rate and Fixed Ratio consistently produced the highest. While absolute adverse event magnitudes scale predictably with the probability parameters (e.g., +25% AE probabilities produce approximately +25% higher absolute rates), the relative ordering and the conclusion that Dynamic Escalation outperforms Fixed Ratio by approximately 35–40% is invariant to these assumptions. The one configuration warranting note is the −25% threshold variation (threshold = 0.660): under this setting, all three SOPs converge to near-identical adverse event rates (~10.9–11.0/100 pd) because the lower threshold causes even Dynamic Escalation to trigger overload classification for most of its operational range, eliminating its workload management advantage. This boundary condition, which requires an implausibly low overload threshold, is the only scenario under which the SOP ranking could be challenged.

This analysis was specifically motivated by the recognition that these three parameters are calibrated from the external literature rather than derived from MIMIC-IV, and therefore carry greater epistemic uncertainty than the empirically fitted patient flow parameters.

In addition to a one-at-a-time sensitivity analysis of the adverse-event probability parameters (Table 10), a joint sensitivity analysis was conducted varying the escalation trigger and overload threshold simultaneously (Table 11), since these two parameters jointly determine Dynamic Escalation’s behavior. Across all six valid threshold combinations tested (escalation ≤ overload), Dynamic Escalation retained the lowest baseline adverse event rate, ranging from 3.10 to 5.16 per 100 patient days depending on configuration, confirming that the SOP ranking in the primary analysis is not an artifact of the specific 0.85/0.88 threshold pairing selected. This ranking stability should be interpreted alongside the resource-constrained finding in Section 3.5: it demonstrates that Dynamic Escalation’s advantage over the static protocols is threshold-stable within the primary (unconstrained) comparison, not that the underlying decision logic outperforms static allocation independent of resource capacity.

A second sensitivity analysis assessed the robustness of the Dynamic Escalation protocol to the instantaneous activation assumption. On-call nurse activation delays of 0, 0.5, 1.0, and 2.0 h were simulated across 100 replications under baseline conditions, using the frozen recalibrated model (Table 12). Adverse event rates increased modestly with activation delay, from 4.260 per 100 patient days at instantaneous activation to 4.350 (0.5 h), 4.406 (1.0 h), and 4.497 (2.0 h). Even under the most conservative 2 h delay assumption, Dynamic Escalation (4.497 per 100 patient days) outperformed Fixed Ratio (5.119 per 100 patient days) by 12.1% in the primary, unconstrained comparison, confirming that this advantage is not an artifact of the instantaneous-activation assumption. As established in Section 3.4 and Section 3.5, this advantage reflects Dynamic Escalation’s access to additional total staffing capacity rather than superior decision logic; the delay sensitivity analysis therefore confirms the robustness of the resource effect to activation latency, not the robustness of the decision logic itself.

### 3.5. Resource-Constrained Comparison Results

Under the resource-constrained design, the SOP ranking changes materially compared to the primary analysis, but not in the direction that would validate Dynamic Escalation’s decision logic. At baseline, Fixed Ratio (5.119 ± 0.568 per 100 patient days) and Acuity-Adjusted (5.221 ± 0.609) were statistically indistinguishable (Dunn’s post hoc, *p* = 0.270, r = 0.090), while Dynamic Escalation under equal resource conditions (6.488 ± 0.611) performed significantly worse than both (vs. Fixed Ratio: *p* < 0.0001, r = −0.897; vs. Acuity-Adjusted: *p* < 0.0001, r = −0.855). This pattern held consistently across all four demand scenarios (Table 13), with the constrained Dynamic Escalation configuration producing the highest adverse event rate under Census Surge, Nurse Shortage, and Combined Disruption.

This result indicates that Dynamic Escalation’s 16.8% advantage over Fixed Ratio in the primary analysis (Table 4) is attributable entirely to its access to greater total staffing capacity, up to 12 day/10 night nurses when escalated, compared to a fixed 10 day/8 night for the other two protocols, rather than to any independent benefit of workload-triggered decision logic. When total nurse hours are held equal, reducing baseline staffing and waiting for a workload threshold to trigger on-call activation performs worse than simply maintaining stable, adequate staffing from the outset (Fixed Ratio) or allocating that same fixed capacity by acuity (Acuity-Adjusted). The reactive nature of the escalation mechanism, i.e., activating only after workload has already crossed 85% of capacity, means that patients experience a period of elevated risk before additional capacity arrives, a cost that is not offset by any compensating benefit once total resources are equalized.

## 4. Discussion

All findings discussed in this section are model-generated outputs produced under specific parameterization assumptions. They should be interpreted as directional, simulation-derived evidence for hypothesis generation and protocol design guidance, and should not be interpreted as direct predictions of clinical outcomes in real ICU populations, where confounding factors, implementation variability, organizational context, and patient heterogeneity would influence the observed results in ways the present model does not capture.

### 4.1. Research Questions Addressed

This study evaluated three ICU nurse staffing SOPs across four demand scenarios using a Discrete Event Simulation model parameterized from 48,495 real ICU stays in MIMIC-IV. The results provide clear, statistically robust answers to the study’s three research questions.

RQ1: Which SOP configuration produces the best patient safety outcomes under normal operating conditions? Dynamic Escalation consistently produced the lowest adverse event rates across all conditions, achieving 4.260 adverse events per 100 patient days at baseline, a 16.8% reduction relative to the Fixed Ratio protocol (5.119) and an 18.4% reduction relative to the Acuity-Adjusted protocol (5.221). These differences were statistically significant (*p* < 0.0001), with moderate-to-large effect sizes (Dynamic Escalation vs. Fixed Ratio: r = −0.745; Dynamic Escalation vs. Acuity-Adjusted: r = −0.781).

However, this primary-analysis advantage reflects differences in total staffing capacity rather than in decision logic. A resource-constrained comparison (Section 2.8, Table 13) equalized maximum nurse hours across all three SOPs by reducing Dynamic Escalation’s base staffing to 8 day/6 night nurses, such that on-call activation restores capacity to the same 10 day/8 night level as Fixed Ratio and Acuity-Adjusted. Under this equal-resource design, Dynamic Escalation no longer outperforms the static protocols, it performs significantly worse than both Fixed Ratio (6.488 vs. 5.119 per 100 patient-days; *p* < 0.0001, r = −0.897) and Acuity-Adjusted (6.488 vs. 5.221; *p* < 0.0001, r = −0.855), which were themselves statistically indistinguishable from each other (*p* = 0.270, r = 0.090).

This indicates that Dynamic Escalation’s entire primary-analysis advantage is attributable to its access to greater total staffing capacity, not to any independent benefit of workload-triggered escalation logic. If anything, the reactive design is a liability with equal resources: reducing baseline staffing and waiting for a workload threshold to be crossed before activating additional nurses leaves patients exposed to a period of elevated risk that static, adequately staffed protocols avoid entirely. The primary analysis (Table 4) should therefore be interpreted as a comparison of complete staffing system configurations that differ in total available capacity, not as evidence that reactive, threshold-based escalation is a superior allocation strategy relative to static assignment when resources are held constant.

RQ2: How do SOP configurations perform under demand disruption? All three SOPs showed statistically significant differences in adverse event rates across every demand scenario (Kruskal–Wallis H = 104.1–122.7, *p* < 0.0001; Table 8), and each SOP’s own adverse event rate varied significantly across scenarios (Fixed Ratio H = 34.4; Acuity-Adjusted H = 26.9; Dynamic Escalation H = 30.1; all *p* < 0.0001), indicating that none of the three protocols operates near a structural capacity ceiling under the modeled conditions (Table 5). This ranking was stable across the joint sensitivity analysis when varying the escalation and overload thresholds simultaneously (Table 11), as well as under activation-delay sensitivity testing (Table 12), confirming that Dynamic Escalation’s advantage in the primary, unconstrained comparison is not an artifact of specific parameter or timing assumptions, although, as established in Section 3.5, this advantage reflects staffing capacity rather than decision logic. Even under Combined Disruption, Dynamic Escalation’s adverse event rate (4.701 per 100 patient days) remained below Fixed Ratio’s own baseline rate (5.119), an 8.2% reduction.

RQ3: Does nurse workforce shortage or patient census surge pose a greater systemic risk? At equal (14-day) duration, this depends on the staffing protocol rather than holding as a general finding. Fixed Ratio and Acuity-Adjusted showed no reliable difference between the two disruption types (Table 7), while Dynamic Escalation showed shortages caused modestly greater degradation than surges. This protocol-specific pattern is consistent with the mechanism by which each disruption acts: census surge increases patient volume while nurse capacity remains intact, whereas workforce shortage directly reduces the capacity that workload-responsive protocols depend on to absorb demand, a mechanism that only manifests where such responsive capacity exists. We therefore do not find support for a general claim that workforce shortage is more harmful than census surge across ICU staffing protocols, and restrict this conclusion to Dynamic Escalation specifically.

### 4.2. Practical Deployment Recommendations

The simulation results support the following directional, simulation-based observations for ICU staffing SOP design. All suggestions should be interpreted as directional guidance rather than prescriptive policy, as real-world implementation depends on organizational culture, staffing regulations, labor agreements, financial constraints, and workforce availability, all of which are factors not incorporated in the present simulation and which must be addressed through implementation science research before clinical translation. For example, mandatory nurse-to-patient ratio legislation in some jurisdictions may constrain the flexibility required for Dynamic Escalation’s threshold-based activation mechanism. Labor agreements governing on-call obligations and overtime compensation will directly affect the feasibility and cost of maintaining an available on-call nurse pool. These contextual factors mean that the optimal protocol configuration identified under simulated conditions may differ from the optimal configuration in any specific real-world institutional setting.

Simulation findings indicate that Dynamic Escalation’s apparent safety advantage over static protocols (16.8% relative to Fixed Ratio in the primary analysis) is attributable predominantly to its access to additional total nurse hours via on-call activation, rather than to a superior underlying allocation rule (Section 3.5, Table 13) [18]. We therefore do not recommend Dynamic Escalation’s threshold-triggered decision logic on safety grounds independent of staffing capacity: its benefit is functionally equivalent to increasing total nurse staffing, and the reactive design does not outperform simply maintaining adequate staffing levels or an acuity-based allocation rule when total capacity is held constant. The on-call activation threshold of 85% workload capacity remains operationally straightforward to implement using existing NAS-based workload monitoring tools [6] for institutions adopting this model for scheduling flexibility, independent of the decision-logic finding reported here.

Fixed Ratio protocols should be treated as a minimum floor, not a staffing target. The New York State 2023 ICU staffing rule requires a minimum 1:2 ratio, increased as appropriate for patient acuity [4], yet the results demonstrate that a pure fixed-ratio implementation produces the highest adverse event rates among the three primary-analysis configurations tested, with a workload index of 0.764 at baseline (Table 5), consistently elevated relative to the other protocols, though not near a structural capacity ceiling [19].

It is worth noting that Law et al. [20] found no detectable patient outcome improvement from acuity-tool-guided staffing ratios in a real-world hospital setting, which appears to contrast with this paper’s simulation findings. The authors themselves acknowledged multiple contributing factors, including that staffing levels may have been adequate prior to the mandate and that hospitals had significant leeway in acuity tool selection and deployment. This discrepancy underscores the gap between protocol design and real-world implementation, a limitation that simulation-based evaluation cannot address, and highlights the need for implementation science research alongside SOP design optimization.

Workforce retention should be treated as a patient safety intervention. The finding that nurse workforce shortage causes greater modeled performance degradation than census surge held only for Dynamic Escalation at equal (14-day) duration; Fixed Ratio and Acuity-Adjusted showed no reliable difference between the two disruption types (Section 3.2, Table 7). This suggests workforce retention may be a more consequential patient safety lever specifically where on-call or reserve-capacity staffing is used, rather than a general finding across all ICU staffing approaches [21]. It should be noted, however, that this study evaluates shortage scenarios rather than directly evaluating retention interventions. The inference that workforce retention functions as a patient safety intervention therefore warrants direct empirical investigation rather than being treated as an established finding. Burnout, chronic fatigue, and occupational stress have been identified as primary drivers of nursing attrition [22], and future work should examine whether interventions targeting these factors produce measurable improvements in safety-relevant staffing outcomes.

Bed overflow is a capacity problem, not a staffing problem. The finding that overflow counts were identical across all three SOPs within each scenario indicates that no staffing protocol, however well-designed, can resolve structural capacity shortfalls. ICUs experiencing high diversion rates require capacity expansion solutions alongside staffing optimization. This finding highlights a fundamental operational distinction: nurse staffing protocols and bed capacity management address different bottlenecks within the ICU system, and optimizing one cannot compensate for deficiencies in the other, a systems-level insight with direct implications for hospital capacity planning.

### 4.3. Limitations and Future Work

This study has several limitations that should be considered when interpreting the results. The most significant is the absence of external predictive validation. This study demonstrates two levels of model credibility: parameter verification: confirming that the implemented distributions accurately reproduce the MIMIC-IV source statistics (Table 3); and internal consistency: demonstrated by stable output distributions across 100 replications (CV < 4%). What has not been demonstrated is external predictive validity, or the capacity of the model to reproduce patient flow behavior and safety outcomes in ICU settings other than BIDMC. Parameterization fidelity was assessed against the same MIMIC-IV data used to derive model inputs, which confirms implementation correctness but does not constitute validation in independent settings. Researchers applying these findings to other ICU contexts should treat the results as directional, simulation-derived evidence rather than transferable predictions.

Second, nursing task demand rates and adverse event probability thresholds were calibrated from the NAS-based literature rather than extracted directly from MIMIC-IV. As a result, absolute adverse event rates reported in this study are conditional on these assumed constants and should not be interpreted as estimates of actual clinical adverse event frequencies. Conclusions regarding the relative ranking of SOP configurations are more robust to this uncertainty, as confirmed by the ±25% sensitivity analysis in Section 3.4, which demonstrates that the SOP ranking is invariant across all tested parameter variations.

Third, patient acuity in the model is assigned probabilistically at arrival based on the empirical distribution of the first-24 h maximum SOFA scores observed in the MIMIC-IV cohort. In real ICU operations, this value is not prospectively available at the moment of admission; it is only known retrospectively after 24 h of clinical observation. The Acuity-Adjusted and Dynamic Escalation protocols therefore operate in the model with idealized advance knowledge of eventual patient acuity, which may overstate the precision achievable by acuity-based staffing rules at the point of admission in real clinical settings, where initial triage relies on presenting severity indicators rather than a retrospective summary score.

Fourth, the model does not capture several important human and organizational factors including nurse experience heterogeneity, skill mix variation, cumulative fatigue accumulation, teamwork quality, communication patterns, and broader organizational culture. These factors are likely to both amplify the adverse effects of prolonged shortage scenarios and moderate the benefits of adaptive staffing protocols in ways the present model cannot reproduce. Their omission means the model represents an idealized version of ICU nursing operations and should not be expected to capture the full complexity of real clinical environments. More specifically, the adverse event generation mechanism reduces a multifactorial clinical phenomenon to a binary Bernoulli process conditioned solely on whether the unit-level workload index exceeds a fixed threshold. This abstraction, while necessary for model tractability and consistent with the shift-level measurement scale of the NAS instrument, cannot capture the within-shift temporal dynamics of error accumulation, the differential vulnerability of patients at different acuity levels to nurse-sensitive harm, or the protective effects of teamwork and communication quality. Additionally, the workload–risk relationship is modeled as a binary threshold, elevated adverse-event probability applies uniformly once workload index exceeds 0.88, with no risk gradient below that point, rather than as a continuous function of workload, creating an artificial discontinuity at the threshold boundary that may not reflect the true, likely more gradual, relationship between nursing workload and adverse-event risk.

Absolute adverse event rates should therefore be interpreted as relative indicators of workload-driven risk rather than as estimates of actual clinical event frequencies.

Similarly, the workload index captures direct patient care task demand only and does not account for the substantial indirect workload components of ICU nursing practice, including documentation burden, family communication, medication preparation, mentoring of junior staff, participation in multidisciplinary rounds, and response to unexpected clinical deterioration or simultaneous emergencies. Real ICU nursing workload is therefore substantially higher than the model represents, meaning the model likely underestimates the cognitive load experienced by nurses, particularly under shortage conditions, and may consequently understate the safety consequences of staffing shortfalls.

Fifth, the on-call nurse activation mechanism in SOP 3 assumes instantaneous response, which may overestimate real-world effectiveness where call-in latency introduces delays. A sensitivity analysis introducing activation delays of 0.5, 1.0, and 2.0 h (Section 3.4) demonstrates that Dynamic Escalation maintains its advantage over Fixed Ratio even at a 2 h delay (4.497 vs. 5.119 adverse events per 100 patient days; −12.1%), though the magnitude of advantage narrows with increasing latency. In practice, the effectiveness of Dynamic Escalation will depend on the availability of a sufficiently large and geographically accessible on-call nurse pool, which varies across institutions.

Sixth, the simulation model was parameterized from a single academic medical center (BIDMC, Boston), and the MIMIC-IV-derived patient flow parameters, including arrival rates, LOS distributions, and acuity proportions, may not generalize to community hospitals, rural ICUs, or non-US healthcare systems with different patient mix and operational structures. Multi-site parameterization using data from diverse ICU types is recommended as a priority for future work.

Beyond geographic and institutional generalizability, the fixed 20-bed configuration represents a median adult MICU size and may not reflect the operational dynamics of smaller community ICUs (typically 8–12 beds), where fixed staffing ratios represent a larger proportion of total available capacity, or larger academic units (30–40 beds), where economies of scale may alter the relative advantage of adaptive protocols. Specialty ICUs, including cardiac, neonatal, neurological, and surgical units, have substantially different acuity profiles, task demand rates, and nurse-to-patient ratio norms that would require unit-specific parameterization before the present findings could be applied. The fixed shift pattern (day 07:00–18:00, night 18:00–07:00) similarly reflects BIDMC’s operational structure and may not generalize to ICUs using 12 h shifts or flexible scheduling arrangements.

Future work should extend this framework in several directions. Multi-site parameterization using data from diverse ICU types, including community hospitals, rural ICUs, and specialty units, would improve generalizability and address the single-site limitation of the present study. Incorporating nurse fatigue accumulation as a time-varying parameter would improve the realism of shortage scenario modeling, particularly for extended workforce shortage conditions. Economic analysis quantifying the cost per adverse event avoided under each SOP configuration, accounting for the incremental on-call staffing hours associated with Dynamic Escalation, would provide the cost-effectiveness framework necessary to support adoption decisions. A full probabilistic sensitivity analysis varying all model inputs simultaneously, including arrival distributions, LOS distributions, acuity proportions, and staffing levels, would provide a more comprehensive characterization of model uncertainty than the targeted parameter analysis conducted here. Finally, integration with real-time NAS-based workload monitoring systems would enable prospective, rather than retrospective, SOP evaluation as part of clinical operations.

## 5. Conclusions

To the authors’ knowledge, this study presents one of the first systematic, simulation-based comparison of ICU nurse staffing SOP robustness under disruption across multiple demand disruption scenarios, using real clinical data from 48,495 ICU stays in MIMIC-IV. Three key conclusions emerge from the results.

First, under the modeled conditions, Dynamic Escalation produced lower adverse event rates than both Fixed Ratio and Acuity-Adjusted in the primary analysis, with a 16.8% reduction relative to Fixed Ratio. However, a resource-constrained comparison equalizing total nurse hours across all three SOPs shows that this advantage does not reflect superior decision logic: under equal resources, Dynamic Escalation performed significantly worse than both static protocols (*p* < 0.0001), which were themselves statistically indistinguishable from one another. This indicates that Dynamic Escalation’s primary-analysis advantage is attributable to its access to greater total staffing capacity via on-call activation, not to any independent benefit of workload-triggered escalation logic. The practical implication is that increasing total nurse staffing capacity, rather than adopting a specific reactive allocation rule, is likely the primary driver of the safety benefits observed under Dynamic Escalation in the primary analysis.

Second, whether nurse workforce shortage poses greater risk than census surge depends on the staffing protocol rather than holding universally: at equal duration, Fixed Ratio and Acuity-Adjusted showed no reliable difference between the two disruption types, while Dynamic Escalation showed that shortages caused modestly greater degradation. This finding should not be generalized as a claim that workforce retention is a more important patient safety lever than surge-capacity planning across all staffing approaches.

Third, the SOP performance ranking is robust to ±25% variation in the three literature-derived adverse event parameters, confirming that the relative conclusions are not dependent on specific calibration assumptions, with one boundary condition identified at implausibly low overload threshold values.

Together, these findings provide quantitative, simulation-based evidence to inform ICU staffing policy discussions and contribute a reusable simulation framework, fully parameterized from open-access clinical data, that can be adapted to evaluate alternative staffing configurations in other ICU settings.

## Figures and Tables

**Table 1:** Simulation model input parameters derived from MIMIC-IV v3.1 (*n* = 48,495 ICU stays).

Parameter	Value	Source	Source Category
ICU bed capacity	20 beds	Literature [14]	Literature-defined
Base nurse—day shift (07:00–18:00)	10	Literature [6]	Literature-defined
Base nurses—night shift (18:00–07:00)	8	Literature [6]	Literature-defined
Arrival rate—day shift	0.121 patients/h	MIMIC-IV	Empirical (MIMIC-IV)
Arrival rate—night shift	0.205 patients/h	MIMIC-IV	Empirical (MIMIC-IV)
LOS—low acuity (μ, σ)	3.611, 1.018	MIMIC-IV	Empirical (MIMIC-IV)
LOS—moderate acuity (μ, σ)	4.249, 0.969	MIMIC-IV	Empirical (MIMIC-IV)
LOS—high acuity (μ, σ)	4.673, 0.848	MIMIC-IV	Empirical (MIMIC-IV)
Acuity distribution (low/moderate/high)	64.4%, 27.4%, 8.2%	MIMIC-IV	Empirical (MIMIC-IV)
Acuity-adjusted staffing ratio—low acuity	1:3	[6,15]	Literature-calibrated (NAS)
Acuity-adjusted staffing ratio—moderate acuity	1:2	[6,15]	Literature-calibrated (NAS)
Acuity-adjusted staffing ratio—high acuity	1:1	[6,15]	Literature-calibrated (NAS)
Adverse event probability—normal workload	0.008 per 8 h shift check	[15,16]	Literature-calibrated (NAS)
Adverse event probability—overloaded	0.035 per 8 h shift check	[15,16]	Literature-calibrated (NAS)
Workload overload threshold	0.88	[6,15]	Literature-calibrated (NAS)
Escalation trigger threshold (SOP 3)	0.85	[6]	Literature-calibrated (NAS)
Maximum bed wait before diversion	2 h	Literature [16]	Literature-defined

**Table 2:** Summary of key modeling assumptions, their rationale, data source, and potential directional impact on results.

Assumption	Rationale	Source	Potential Impact on Results
Poisson patient arrival process	Standard DES assumption for independent random arrivals	MIMIC-IV timestamps	May underestimate burst arrivals during acute surge events
Log-normal LOS distributions	Skewed positive distribution matching empirical LOS data	MIMIC-IV LOS fit	May over/underestimate extreme length stays
Binary Bernoulli adverse event model	Tractable abstraction consistent with NAS shift-level measurement scale	NAS literature	Underestimates multifactorial adverse event etiology
8 h shift adverse event check	Matches NAS measurement interval	NAS framework	Misses intra-shift temporal clustering of errors
Instantaneous on-call activation	Simplifying assumption; tested via delay sensitivity analysis	Modeling convention	Overestimates Dynamic Escalation advantage; robustness confirmed at 2 h delay
Fixed 20-bed capacity	Median adult MICU size	Literature [14]	May not generalize to small or large ICUs
Fixed 2-shift pattern	Reflects BIDMC operational structure	Modeling convention	May not generalize to 12 h shift ICUs
2 h diversion threshold	Standard ICU boarding practice	Literature [14]	Attenuates full surge volume; structural shielding effect acknowledged
Workload index based on task demand only	Tractable proxy for nursing workload	NAS literature	Underestimates true cognitive load; documented as limitation

**Table 3:** Empirical validation: simulated baseline output vs. MIMIC-IV cohort statistics.

Metric	MIMIC-IV	Simulated	Error (%)
Mean LOS—low acuity (h)	62.12	62.12	0.0%
Median LOS—low acuity (h)	37.00	37.00	0.0%
Mean LOS—moderate acuity (h)	111.96	111.96	0.0%
Median LOS—moderate acuity (h)	70.00	70.00	0.0%
Mean LOS—high acuity (h)	153.23	153.23	0.0%
Median LOS—high acuity (h)	107.00	116.57	8.9%
Overall mean LOS (h)	83.25	83.25	0.0%
Daily admissions	3.99	3.73	6.5%
Bed Occupancy Rate	68.9%	68.9%	0.0%

**Table 4:** Simulation results: mean ± SD across 100 replications per SOP–scenario combination. AE = adverse event; pd = patient days.

SOP	Scenario	AE Rate (Mean ± SD [95% CI])	Service Level	Workload Index	Overflow (pts/yr)
Fixed Ratio (1:2)	Baseline	5.119 ± 0.568 [5.008–5.230]	0.9839	0.7643	27
Fixed Ratio (1:2)	Census Surge	5.360 ± 0.649 [5.233–5.488]	0.9832	0.7777	37
Fixed Ratio (1:2)	Nurse Shortage	5.422 ± 0.574 [5.310–5.535]	0.9830	0.7773	27
Fixed Ratio (1:2)	Combined Disruption	5.648 ± 0.642 [5.522–5.774]	0.9823	0.7880	37
Acuity-Adjusted	Baseline	5.221 ± 0.609 [5.102–5.340]	0.9836	0.7511	27
Acuity-Adjusted	Census Surge	5.439 ± 0.722 [5.297–5.580]	0.9829	0.7643	37
Acuity-Adjusted	Nurse Shortage	5.503 ± 0.626 [5.381–5.626]	0.9827	0.7640	27
Acuity-Adjusted	Combined Disruption	5.734 ± 0.708 [5.595–5.873]	0.9820	0.7758	37
Dynamic Escalation	Baseline	4.260 ± 0.478 [4.167–4.354]	0.9866	0.7202	27
Dynamic Escalation	Census Surge	4.394 ± 0.553 [4.286–4.503]	0.9862	0.7317	37
Dynamic Escalation	Nurse Shortage	4.484 ± 0.515 [4.383–4.585]	0.9859	0.7312	27
Dynamic Escalation	Combined Disruption	4.701 ± 0.577 [4.588–4.815]	0.9853	0.7430	37

**Table 5:** Workload index (mean ± SD across 100 replications) per SOP and demand scenario. Range = max WI − min WI across all four scenarios.

SOP	Baseline	Census Surge	Nurse Shortage	Combined	Range (Max − Min)
Fixed Ratio (1:2)	0.7643 ± 0.0242	0.7777 ± 0.0234	0.7773 ± 0.0236	0.7880 ± 0.0227	0.0237
Acuity-Adjusted	0.7511 ± 0.0272	0.7643 ± 0.0291	0.7640 ± 0.0266	0.7758 ± 0.0282	0.0247
Dynamic Escalation	0.7202 ± 0.0233	0.7317 ± 0.0246	0.7312 ± 0.0231	0.7430 ± 0.0241	

**Table 6:** Adverse event rate during disruption windows, absolute change from pre-disruption baseline.

SOP	Scenario	Pre-Disruption	During Disruption	Post-Disruption	Δ (During − Pre)
Fixed Ratio	Census Surge (14 d)	5.467	8.571	5.151	+3.104
Acuity-Adjusted	Census Surge (14 d)	5.620	8.118	5.246	+2.498
Dynamic Escalation	Census Surge (14 d)	4.557	6.226	4.256	+1.669
Fixed Ratio	Nurse Shortage (30 d)	5.467	8.427	5.069	+2.960
Acuity-Adjusted	Nurse Shortage (30 d)	5.620	8.204	5.167	+2.584
Dynamic Escalation	Nurse Shortage (30 d)	4.557	6.574	4.226	+2.017
Fixed Ratio	Combined (14 d acute)	5.467	11.206	5.365	+5.739
Acuity-Adjusted	Combined (14 d acute)	5.620	11.004	5.455	+5.384
Dynamic Escalation	Combined (14 d acute)	4.557	9.784	4.441	+5.227

Note: “Pre-Disruption” and “Post-Disruption” reflect the mean adverse event rate outside the disruption window within the same 365-day replication; “During Disruption” is computed only over the actual 14- or 30-day disruption period, avoiding annualized dilution of the effect.

**Table 7:** Symmetric scenario comparison: adverse event rate increase from baseline under 14-day census surge vs. 14-day and 30-day nurse shortage.

SOP	Δ Census Surge (14 d)	Δ Nurse Shortage (14 d)	Δ Nurse Shortage (30 d)	Finding
Fixed Ratio	+3.104	+3.054	+2.960	No reliable difference at equal duration
Acuity-Adjusted	+2.498	+2.503	+2.584	No reliable difference at equal duration
Dynamic Escalation	+1.669	+1.777	+2.017	Shortage modestly worse

**Table 8:** Kruskal–Wallis test results for adverse event rate across SOP configurations per scenario. *** *p* < 0.001.

Scenario	H Statistic	*p*-Value	Significance	Interpretation
Baseline	116.950	<0.001	***	All SOPs differ
Census Surge	107.123	<0.001	***	All SOPs differ
Nurse Shortage	122.685	<0.001	***	All SOPs differ
Combined Disruption	104.106	<0.001	***	All SOPs differ

Note: Kruskal–Wallis tests across demand scenarios within each SOP: Fixed Ratio H = 34.432, *p* < 0.0001 (***); Acuity-Adjusted H = 26.879, *p* < 0.0001 (***); Dynamic Escalation H = 30.127, *p* < 0.0001 (***).

**Table 9:** Dunn’s post hoc pairwise comparisons (Bonferroni-corrected *p*-values) and rank-biserial effect sizes for adverse event rate under baseline and combined disruption scenarios.

Comparison	Baseline (*p*)	Combined Disruption (*p*)	Effect Size (r), Baseline	Effect Size (r), Combined
Fixed Ratio vs. Acuity-Adjusted	0.81 (ns)	1.00 (ns)	0.090	0.070
Fixed Ratio vs. Dynamic Escalation	<0.0001	<0.0001	−0.745	−0.717
Acuity-Adjusted vs. Dynamic Escal.	<0.0001	<0.0001	−0.781	−0.726

**Table 10:** Sensitivity analysis: adverse event rates (mean, 15 replications) under ±25% variation in adverse event probability parameters and overload threshold.

Parameter Variation	Fixed Ration (Baseline)	Acuity-Adj. (Baseline)	Dyn. Escal. (Baseline)	Fixed Ratio (Combined)	Acuity-Adj. (Combined)	Dyn. Escal. (Combined)
Base (0.008/0.035/0.88)	5.183	5.286	4.301	5.840	5.913	4.820
+25% AE probs (0.010/0.044)	6.459	6.595	5.378	7.284	7.409	6.053
−25% AE probs (0.006/0.026)	3.870	3.940	3.222	4.323	4.402	3.600
+25% Threshold (0.95, capped)	4.440	4.534	3.542	5.087	5.162	4.027
−25% Threshold (0.66)	8.594	8.518	8.392	9.028	8.901	8.786

Note: A literal +25% multiplicative increase from the base overload threshold (0.88 × 1.25 = 1.10) exceeds the maximum possible workload index, which is capped at 1.0 by construction (Section 2.4), and would therefore never trigger overload classification under any modeled condition. We instead report the highest threshold value within the model’s feasible range (0.95) as the upper sensitivity bound.

**Table 11:** Joint Sensitivity Analysis: adverse event rates (mean, 30 replications per configuration) under combined variation in the Dynamic Escalation trigger threshold and the overload threshold, baseline scenario. Only configurations where the escalation threshold does not exceed the overload threshold are reported, as an escalation trigger above the overload point would activate on-call staffing only after adverse-event risk has already increased.

Escalation	Overload	Fixed Ratio	Acuity-Adjusted	Dynamic Escalation
0.75	0.80	6.937	6.470	5.157
0.75	0.88	5.183	5.286	3.782
0.75	0.95	4.440	4.534	3.102
0.85	0.88	5.183	5.286	4.301
0.85	0.95	4.440	4.534	3.542
0.95	0.95	4.440	4.534	3.756

**Table 12:** Activation Delay Sensitivity Analysis: Dynamic Escalation adverse event rate under instantaneous and delayed on-call nurse activation (100 replications, baseline scenario). Reference: Fixed Ratio baseline AE rate = 5.119 per 100 patient days.

Activation Delay (Hours)	Dynamic Escalation AE Rate (per 100 pd)	SD	Vs. No Delay	Vs. Fixed Ratio
0 (instantaneous)	4.260	0.475	-	−16.8%
0.5	4.350	0.491	+2.1%	−15.0%
1.0	4.406	0.502	+3.4%	−13.9%
2.0	4.497	0.504	+5.6%	−12.1%

**Table 13:** Resource-constrained comparison: adverse event rates (mean ± SD, 100 replications) under equal maximum nurse hours. Dynamic Escalation base staffing reduced to eight day/six night nurses; on-call activation restores capacity to 10 days/8 nights, equal to Fixed Ratio and Acuity-Adjusted base staffing.

SOP	Baseline	Census Surge	Nurse Shortage	Combined Disruption
Fixed Ratio	5.119 ± 0.568	5.360 ± 0.649	5.422 ± 0.574	5.648 ± 0.642
Acuity-Adjusted	5.221 ± 0.609	5.439 ± 0.722	5.503 ± 0.626	5.734 ± 0.708
Dynamic Escalation (Constrained)	6.488 ± 0.611	6.682 ± 0.713	6.690 ± 0.605	6.863 ± 0.710

## Data Availability

Restrictions apply to the availability of these data. Data were obtained from PhysioNet and are available at https://physionet.org/content/mimiciv (accessed on 2 June 2026) with the permission of PhysioNet.

## Abbreviations

The following abbreviations are used in this manuscript:

AE	Adverse Event
BIDMC	Beth Israel Deaconess Medical Center
DES	Discrete Event Simulation
EHR	Electronic Health Record
ICU	Intensive Care Unit
LOS	Length of Stay
MIMIC-IV	Medical Information Mart for Intensive Care, Version IV
MICU	Medical Intensive Care Unit
SICU	Surgical Intensive Care Unit
NAS	Nursing Activity Score
Pd	Patient days
RN	Registered Nurse
SEIPS	Systems Engineering Initiative for Patient Safety
SOFA	Sequential Organ Failure Assessment
STRESS	Strengthening the Reporting of Empirical Simulation Studies
WI	Workload Index
